# The effect of three novel feature extraction methods on the prediction of the subcellular localization of multi-site virus proteins

**DOI:** 10.1080/21655979.2017.1373536

**Published:** 2017-11-22

**Authors:** Lei Wang, Yaou Zhao, Yuehui Chen, Dong Wang

**Affiliations:** a School of Information Science and Engineering, University of Jinan, Jinan, China; b Shandong Provincial Key Laboratory of Network Based Intelligent Computing, Jinan, China

**Keywords:** feature extraction, I-PseAAC, PseAAC2, R-Dipeptide, subcellular localization

## Abstract

Experimental methods play a crucial role in identifying the subcellular localization of proteins and building high-quality databases. However, more efficient, automated computational methods are required to predict the subcellular localization of proteins on a large scale. Various efficient feature extraction methods have been proposed to predict subcellular localization, but challenges remain. In this paper, three novel feature extraction methods are established to improve multi-site prediction. The first novel feature extraction method utilizes repetitive information via moving windows based on a dipeptide pseudo amino acid composition method (R-Dipeptide). The second novel feature extraction method utilizes the impact of each amino acid residue on its following residues based on pseudo amino acids (I-PseAAC). The third novel feature extraction method provides local information about protein sequences that reflects the strength of the physicochemical properties of residues (PseAAC2). The multi-label k-nearest neighbor algorithm (MLKNN) is used to predict the subcellular localization of multi-site virus proteins. The best overall accuracy values of R-Dipeptide, I-PseAAC, and PseAAC2 when applied to dataset S from Virus-mPloc are 59.92%, 59.13%, and 57.94% respectively.

## Introduction

Knowledge about the subcellular localization of proteins is critical for understanding their functions and biological processes in cells.^1^ High-quality databases of information on the subcellular localization of proteins are informed by wet laboratory experiments. However, such experiments are time-consuming, costly and laborious.^2^ Experimental methods for handling proteins on a large scale have become increasingly difficult. It is necessary to develop effective computational methods to analyze subcellular localization.^3^ The web servers proposed to identify the subcellular localization of proteins based on their sequence information can be classified into two series.^4^ One is the “PLoc” series, and the other is the “iLoc” series. The “PLoc” series includes six web servers to handle eukaryotic, plant, human, gram-negative bacterial, gram-positive bacterial, and viral proteins, while the “iLoc” series includes seven web servers to handle eukaryotic, plant, human, animal, gram-negative bacterial, gram-positive bacterial, and viral proteins.^5^ Many studies have indicated that greater progress in prediction systems is obtained by developing feature extraction methods than by improving the classifiers.^6,7^

In recent years, a wide range of feature extraction methods have been proposed to improve the performance of prediction: (1) amino acid composition (AAC) methods;^8-11^ (2) homology-based methods;^7,12^ (3) sorting signal-based methods;^13-14^ and (4) pseudo amino acid-based feature methods (PseAAC).^15-17^ All these methods have shown good performance but could be improved. AAC methods lack location information; homology-based methods are not suitable for low-homology protein sequences; and PseAAC can reflect some of the effects of sequence order but lacks the impact of each residue on the subsequent residues. Therefore, three feature extraction methods are proposed to improve the performance of multi-site prediction.

The three novel feature extraction methods proposed in this study are called R-Dipeptide, I-PseAAC and PseAAC2. Inspired by the long short-term memory with attention mechanism (A-LSTM),^18^ R-Dipeptide focuses on using repetitive information. First, the spacing between two windows is set by the user, often to a small number. In this study, the spacing is one. Then, two better protein sub-sequences are selected according to the prediction results and combined. This method makes up for the lack of extraction of key information by PseAAC. I-PseAAC computes the impact of each amino acid residue on the subsequent residues. This method offers global order information, rather than the local order information provided by PseAAC. PseAAC2 focuses on location information. This method not only offers global order information but also adds the relative strengths of the residues, whereas PseAAC lacks information on the relative strengths of residues.

## Material and methods

### Dataset

Dataset S, constructed by Shen in establishing Virus-mPloc, is the benchmark dataset for the study.^19^ Dataset S offers three advantages. (1) The dataset is specialized for virus proteins. (2) None of the proteins included in S has ≥ 25% pairwise sequence identity to any other protein in the same location. (3) The dataset includes proteins with more than one location and thus can be utilized to address the subcellular localization of multi-site virus proteins.^20^

Dataset S includes 207 virus protein sequences, of which 165 belong to one subcellular location, 39 to two locations, and 3 to three locations.^20^ The dataset is classified into 6 subcellular locations,^21^ as expressed in Eq. 1:

(1) S=S1∪S2∪S3∪S4∪S5∪S6

where *S1* represents the subset for the subcellular location “viral capsid”, *S2* the subset for “host cell membrane”, and so forth (Table 1), while∪denotes “union” in set theory.^21^

**Table 1.** The benchmark dataset S taken from Virus-mPloc^21^.

Subset	Subcellular location	Number of proteins
*S1*	Host viral capsid	8
*S2*	Host cell membrane	33
*S3*	Host endoplasmic reticulum	20
*S4*	Host cytoplasm	87
*S5*	Host nucleus	84
*S6*	Secreted	20
Total number of locative proteins		252
Total number of different proteins		207

Here, the locative protein sequences and different protein sequences are briefly described as follows. Locative proteins are described by Eq. 2:

(2) $$N(locative)=N(different)+\sum_{m=1}^{M}\left(m-1)N(m\right)$$

where *N(locative)* represents the number of locative proteins and *N(different)* represents the number of different proteins. Here, *m* is the number of locations where the specific protein is identified, and *N(m)* is the number of proteins that are identified in *m* locations.

### R-Dipeptide

R-Dipeptide utilizes repetitive information via moving windows based on a dipeptide pseudo amino acid composition method.

First, the number of each amino acid residue in every protein sequence is calculated in Eq. 3. Then, the number of residues is normalized in Eq. 4.

(3) $$V=[v_{1},v_{2},v_{3},...,v_{i},...,v_{20}]$$

where *v_i_* is the number of the *i-th* type of residue in every protein sequence.

(4) $$v_{i}^{*}=\frac{v_{i}-\mu }{\sigma }$$

where $v_{i}^{*}$ is the normalized value of *v_i_*, μ denotes the mean of *v_i_,* and σ represents the standard deviation of *v_i_*.

Second, the spacing between two windows is set to one, and the window size is set to thirty. The sub-sequence of the first group is {R_1_,R_2_,…,R_30_}, the sub-sequence of the second group is {R_2_,R_3_,…,R_31_}, and so forth. For the last residue (R_L_), L is smaller than the minimum length of all protein sequences. Then, two improved protein sub-sequences are combined to create a new database based on the prediction results. The new database contains important repetitive information. that contributes to the prediction of subcellular localization.

Lastly, a dipeptide pseudo amino acid composition method (Dipeptide) is used for the new database. Dipeptide will generate 400 components, i.e., AA, AC, AD, …, YV, YW, and YY. These 400 components are calculated for every protein sequence and then subjected to a standard conversion.

### I-PseAAC

PseAAC is proposed by Chou and avoids losing the ordering information of protein sequences.^23^

A protein (P) including *L* amino acid residues can be described by Eq. 5:

(5) $$P=R_{1},R_{2},R_{3},......,R_{L}$$

where *R_1_* is the first residue of the protein sequence *P, R_2_* is the second residue of the protein sequence *P*, and so forth.

The sequence order information can be represented by Eq. 6.

(6) δθ=∑i=1L−θΩ(Ri,Ri+θ)/(L−θ), (θ=1,2,…,n and n<L) 

where *δ_θ_* is the θ*-th* correlation factor, which provides the sequence order information between the θ most contiguous residues. *Ω(R_i_, R_i+1_)* can be described by Eq. 7:

(7) $$\begin{array}{l} \Omega (R_{i},R_{i+1})=\frac{1}{6}\{[H_{1}(R_{j})-H_{1}(R_{i}){]}^{2}+[H_{2}(R_{j})-H_{2}(R_{i}){]}^{2}+[Pk_{1}(R_{j})-Pk_{1}(R_{i}){]}^{2}\\ +[Pk_{2}(R_{j})-Pk_{2}(R_{i}){]}^{2}+[PI(R_{j})-PI(R_{i}){]}^{2}+[M(R_{j})-M(R_{i}){]}^{2}\} \end{array}$$

where *H_1_(R_i_), H_2_(R_i_), Pk_1_(R_i_), Pk_2_(R_i_), PI(R_i_),* and *M(R_i_)* denote the hydrophobicity value, the hydrophilicity value, *Pk1(-COOH), Pk2(-NH3), PI*, and the mass value of the amino acid residue *R_i_*, respectively.

All physicochemical properties should be normalized before being used in the calculation of Eq. 7.

**Figure 1.**
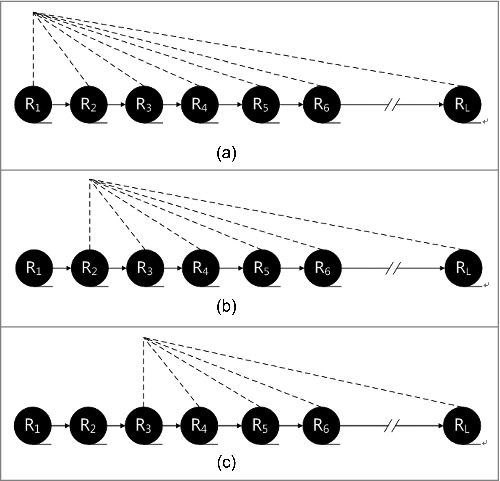
(a) The impact of each residue on the subsequent residues. Fig. 1(b). The impact of each residue on the subsequent residues. Fig. 1(c). The impact of each residue on the subsequent residues.

In contrast to PseAAC, I-PseAAC utilizes the impact of each residue on the subsequent residues. I-PseAAC is described in Fig. 1(a), Fig. 1(b) and Fig. 1(c).

Fig. 1(a), Fig. 1(b) and Fig. 1(c) show the process of I-PseAAC. PseAAC calculates the order information for (R_1_,R_2_), (R_2_,R_3_), (R_3_,R_4_) and so forth, while I-PseAAC calculates the or information for (R_1_,R_2_), (R_1_,R_3_),(R_1_,R_4_) and so forth. The details are shown in Fig. 1(a), Fig. 1(b) and Fig. 1(c). In $\left(R_{i},R_{j}\right)$, *j* is greater than *i*.

### PseAAC2

In contrast to PseAAC and I-PseAAC, PseAAC2 provides a different kind of local information to reflect the strength of the physicochemical properties of residues, as described in Eq. 8 and Eq. 9:

(8) $$\Omega (R_{i})=\frac{1}{6}[H_{1}{\left(R_{i}\right)}^{2}+H_{2}{\left(R_{i}\right)}^{2}+Pk_{1}{\left(R_{i}\right)}^{2}+Pk_{2}{\left(R_{i}\right)}^{2}+PI{\left(R_{i}\right)}^{2}+M{\left(R_{i}\right)}^{2}]$$

(9) $$\Omega (R_{i},R_{j})=\Omega (R_{i})*R(R_{j})$$

### MLKNN

MLKNN is a multi-label classifier that utilizes the k-nearest neighbor algorithm to collect the category tag information of neighbor samples and exploits the principle of maximum posterior probability to infer the “no example of label” set.^21,24^ MLKNN can be described by Eq. 10 and Eq. 11:

(10) $$C_{j}=\sum_{\left(x,Y)\in N(x\right)}\left\{y_{j}\in Y\right\}$$

where *C_j_* represents the number of neighbors of *x* belonging to class *N(x)*.^21^

(11) h(x)={yj|P(Hj|Cj)P(H¬j|Cj)>0.5,1 ≤ j ≤ q}

where *H_j_* denotes the event of *x* including the category *y_j_. P(H_j_|y_j_)* denotes the posterior probability set *H_j_* that *N(x)* contains the number *C_j_* in the category *y_j_.*

### Evaluation

To provide a more intuitive and easier-to-understand measurement, a new scale, the so-called “absolute true” overall accuracy,^20^ reflecting the accuracy of a predictor, is given in Eq. 12:

(12) $$\Lambda =\frac{\sum_{\text{i}=1}^{N}\Delta (i)}{N}$$

where Λ represents the absolute true rate, *N* represents the number of total proteins investigated, and *Δ(i) = 1* or *Δ(i) = 0*.

All subcellular locations of the *i-th* protein will be tested. If every subcellular location of the *i-th* protein is correctly predicted, *Δ(i) = 1*; otherwise, *Δ(i) = 0*.

Therefore, the absolute true scale is much stricter than the scale used previously to measure the overall accuracy.

In addition, a series of other evaluation functions are applied to evaluate the prediction performance.^22^

HammingLoss:

(13) $$Ham\min gLoss(h)=\frac{1}{N}\sum_{i=1}^{N}\frac{1}{C}\left|h(x_{i})\Delta y_{i}\right|$$

HammingLoss is utilized to calculate how many times a label is misclassified. A lower value of HammingLoss represents better algorithm performance.

RankingLoss:

(14) $$RankingLoss(h)=\frac{1}{N}\sum_{i=1}^{N}\frac{1}{\left|C_{i}\right|\left|\bar{C_{i}}\right|}\cdot \left\{\left(y_{1},y_{2})|h(x_{i},y_{1})\;\leq \;h(x_{i},y_{2}\right)\right\}$$

*C_i_* is the collection of labels with a value of one, denoted by labels-one. $\bar{C_{i}}$ is the collection of labels with a value of zero, denoted by labels-zero. If the predictive labels of an instance are completely correct, the output value of labels-one should be higher than the output value in labels-zero. RankingLoss is utilized to calculate how many times the output lacks an appropriate comparison. A lower value of RankingLoss indicates better algorithm performance.

One_error:

(15) $$One\_error(h)=\frac{1}{N}\sum_{i=1}^{N}\left\{[\arg\underset{y\in Y}{\max}h(x_{i},y)]\notin Y_{i}\right\}$$

One_error calculates how many times the top label is not in the appropriate label sets. A lower value of One_error represents better algorithm performance.

Coverage:

(16) $$Coverage(h)=\frac{1}{N}\sum_{i=1}^{N}\frac{\max rank^{h}(x_{i},y)-1}{C}$$

Coverage is utilized to calculate how far down the label set of an instance it is necessary to go. A lower value of Coverage indicates better algorithm performance.

Average_Precision:

(17) $$Average\backslash \_\mathrm{Pr}ecision(h)=\frac{1}{N}\sum_{i=1}^{N}\frac{1}{\left|C_{i}\right|}\sum_{y\in C_{i}}\frac{\left|\left\{y'\in C_{i}|rank^{h}(x_{i},y')\;\leq \;rank^{h}\;(x_{i},y)\right\}\right|}{rank^{h}(x_{i},y)}$$

Average_Precision is utilized to calculate the average fraction of labels ranked. A higher value of Average_Precision represents better algorithm performance.

## Results

In this study, the spacing between two windows is set to 1, and the window size is set to 30. The database is divided into 24 groups: (0,30) is the first group, (1,30) is the second group, and so forth. The number of each amino acid residue in every group is calculated in Eq. 3. and Eq. 4. The overall accuracy of each group is shown in Table 2.

**Table 2.** Sorting signals of database.

(0,30)	(1,30)	(2,30)	(3,30)	(4,30)	(5,30)	(6,30)	(7,30)
50%	45.63%	50%	54.76%	**57.54%**	53.57%	**57.54%**	47.22%
(8,30)	(9,30)	(10,30)	(11,30)	(12,30)	(13,30)	(14,30)	(15,30)
55.56%	46.43%	44.05%	41.27%	49.21%	50%	43.25%	50.79%
(16,30)	(17,30)	(18,30)	(19,30)	(20,30)	(21,30)	(22,30)	(23,30)
50.79%	51.98%	55.56%	56.35%	46.83%	46.03%	46.03%	44.05%

The overall accuracy of the original database is 55.16%, while the best overall accuracy of the groups is 57.54%. Table 2 demonstrates the effect of the sorting-signal method. Group 5 and group 7 are combined to create a new database. For example, group 5 {ACDVY} and group 7 {DVYWY} are converted to the new database {ACDVYDVYWY}. The important repetitive information is {DVY}. If both group 5 and group 7 show good performance, we believe that the two groups share important information {DVY} for the prediction of subcellular localization.

As shown in Table 3, the two methods AAC and Dipeptide give better results when applied to the new database than when applied to the original database. The original database contains redundant information. Therefore, the methods cannot obtain better performance when applied to the original database. The new database utilizes the repetitive information from sub-sequences. This approach is equivalent to increasing the weight of key residues.

**Table 3.** Application of two methods to original database and new database.

AAC in original dataset	R-AAC	Dipeptide in original dataset	R-Dipeptide
55.16%	**58.33%**	54.76%	**59.92%**

Six physicochemical properties are used in the PseAAC2 and I-PseAAC methods: the hydrophobicity, hydrophilicity, *Pk1(-COOH), Pk2(-NH3), PI* and mass values of each amino acid residue, as described in Table 4.

**Table 4.** Six physicochemical properties.

hydrophobicity	hydrophilicity	Pk1	Pk2	PI	mass
0.62	−0.5	2.35	9.87	6.11	15
0.29	−1	1.71	10.78	5.02	47
−0.9	3	1.88	9.6	2.98	59
−0.74	3	2.19	9.67	3.08	73
1.19	−2.5	2.58	9.24	5.91	91
0.48	0	2.34	9.6	6.06	1
−0.4	−0.5	1.78	8.97	7.64	82
1.38	−1.8	2.32	9.76	6.04	57
−1.5	3	2.2	8.9	9.47	73
1.06	−1.8	2.36	9.6	6.04	57
0.64	−1.3	2.28	9.21	5.74	75
−0.78	0.2	2.18	9.09	10.76	58
0.12	0	1.99	10.6	6.3	42
−0.85	0.2	2.17	9.13	5.65	72
−2.53	3	2.18	9.09	10.76	101
−0.18	0.3	2.21	9.15	5.68	31
−0.05	−0.4	2.15	9.12	5.6	45
1.08	−1.5	2.29	9.74	6.02	43
0.81	−3.4	2.38	9.39	5.88	130
0.26	−2.3	2.2	9.11	5.63	107

The three novel feature extraction methods are compared with PseAAC.^23^ Group 5 and group 7 are combined to create a new database, and four feature extraction methods are used in the new database to identify the subcellular localization of multi-site virus proteins by MLKNN. The results of the PseAAC method are obtained via a web server called PseAAC at http://www.csbio.sjtu.edu.cn/bioinf/PseAAC/#. The weight factor is 0.05, and the Lambda parameter is 40.

As shown in Table 5, the three novel feature extraction methods show superior performance, achieving 59.92%, 59.13%, and 57.94% accuracy for the MLKNN algorithm. The PseAAC method shows 57.14% accuracy for MLKNN algorithm. Thus, the three novel feature extraction methods improve the performance of multi-site prediction.

**Table 5.** Application of PseAAC, R-Dipeptide, I-PseAAC and PseAAC2 to the new database.

PseAAC	R-Dipeptide	I-PseAAC	PseAAC2
57.14%	**59.92%**	**59.13%**	**57.94%**

As shown in Table 6, the number of correct predictions of every subcellular location is calculated by Eq. 12. The overall accuracy is the sum of the correct predictions.

**Table 6.** Overall accuracy of R-Dipeptide, I-PseAAC, and PseAAC2.

	Overall accuracy		
Subcellular location	R-Dipeptide	I-PseAAC	PseAAC2
Viral capsid	7/8 = 87.5%	7/8 = 87.5%	7/8 = 87.5%
Host cell membrane	12/33 = 36.36%	13/33 = 39.39%	13/33 = 39.39%
Host endoplasmic reticulum	11/20 = 55%	11/20 = 55%	10/20 = 50%
Host cytoplasm	49/87 = 56.32%	52/87 = 59.77%	51/87 = 58.62%
Host nucleus	59/84 = 70.24%	51/84 = 60.71%	52/84 = 61.9%
Secreted	13/20 = 65%	15/20 = 75%	13/20 = 65%
Overall accuracy	151/252 = 59.92%	149/252 = 59.13%	146/252 = 57.94%

To simplify the representation of the evaluation functions, Average_Precision is denoted by A, Coverage is denoted by C, HammingLoss is denoted by H, One_error is denoted by O, and RankingLoss is denoted by R. The calculation details of the five evaluation functions are described in Eq. 13-17. The feature extraction method is denoted by FEM.

As shown in Table 7, R-Dipeptide, I-PseAAC, PseAAC2 all show better performance than PseAAC in general.

**Table 7.** Evaluation functions for PseAAC, R-Dipeptide, I-PseAAC, and PseAAC2.

FEM	A	C	H	O	R
PseAAC	0.7662	0.1798	0.1428	0.3888	0.1396
R-Dipeptide	0.7838↑	0.1712↓	0.1296↓	0.3571↓	0.1293↓
I-PseAAC	0.7684↑	0.1798	0.1355↓	0.3809↓	0.1396
PseAAC2	0.7686↑	0.1772↓	0.1329↓	0.3849↓	0.1365↓

## Conclusion and discussion

In this study, three novel feature extraction methods are proposed to improve the performance of multi-site prediction. In experimental comparisons, the R-Dipeptide, I-PseAAC, and PseAAC2 methods achieve higher accuracy rates for the MLKNN algorithm than does the PseAAC method. Thus, repetitive information, the impact of each residue on subsequent residues, and local information are critical for the performance of multi-site prediction. The advantage of R-Dipeptide is the extraction of key information using the repetitive information method. We are accustomed to extracting key information by weight adjustment of the algorithm. For a large-scale dataset, weight adjustment is an effective method for the extraction of key information. However, if the dataset is limited in scale, the repetitive information method is better than the weight adjustment method. The advantage of I-PseAAC is that it can reflect the difference in physicochemical properties between each amino acid residue and the subsequent residues. In addition, I-PseAAC provides global information on the residues. The disadvantage is that the difference between the *i-th* residue and the *j-th* residue may be the same as the difference between the *i-th* residue and the *k-th* residue. For example, two kinds of physicochemical properties are denoted by A and B, respectively. The difference in A between the *i-th* residue and the subsequent *j-th* residue is 0.2, and the difference in B is −0.2. The difference between the *i-th* residue and the subsequent *k-th* residue in A is 0.3, and the difference in B is −0.3. Thus, there is no difference between the *j-th* residue and the *k-th* residue. The advantage is that PseAAC2 amplifies the differences in the physicochemical properties of different residues by providing another source of local information about protein sequences. The disadvantage is how to choose a set of representative physicochemical properties. If the values of the physicochemical properties of different residues are different, this kind of physicochemical property is representative. If some of the residues have the same physicochemical property values, the performance of PseAAC2 will decline.

The three novel feature extraction methods have shown good performance but can still be improved. The first question is how to set an appropriate window size and spacing between two windows. If the window is too small, important information will be lost and a large number of groups will be generated. If the window is too large, too much redundant information will be generated. If the spacing between two windows is too large, repeat information will be lost. In addition, groups can be combined in a variety of ways, such as adjacent groups (group 4, group 5), interval groups (group 4, group 7), or more than two groups (group 4, group 5, group 7). Our future studies will focus on these questions with regard to subcellular localization.
